# Phenolic Compounds from *Allium schoenoprasum, Tragopogon pratensis* and *Rumex acetosa* and Their Antiproliferative Effects

**DOI:** 10.3390/molecules16119207

**Published:** 2011-11-03

**Authors:** Zdenka Kucekova, Jiri Mlcek, Petr Humpolicek, Otakar Rop, Pavel Valasek, Petr Saha

**Affiliations:** 1 Polymer Centre, Faculty of Technology, Tomas Bata University at Zlin, T.G.M. sq. 275, 762 72, Zlin, Czech Republic; Email: kucekova@ft.utb.cz (Z.K.); 2 Department of Food Technology and Microbiology, Faculty of Technology, Tomas Bata University at Zlin, T.G.M. sq. 275, 762 72 Zlin, Czech Republic; Email: mlcek@ft.utb.cz (J.M.); rop@ft.utb.cz (O.R.); 3 Centre for Polymer Systems, Polymer Centre, Tomas Bata University at Zlin, T.G.M. sq. 5555, 760 05 Zlin, Czech Republic; Email: valasek@ft.utb.cz (P.V.); saha@utb.cz (P.S.)

**Keywords:** *Allium schoenoprasum*, *Tragopogon pratensis*, *Rumex acetosa*, proliferation, phenolic compounds, HaCaT

## Abstract

Experimental studies have shown that phenolic compounds have antiproliferative and tumour arresting effects. The aim of this original study was to investigate the content of phenolic compounds (PhC) in flowers of *Allium schoenoprasum* (chive), *Tragopogon pratensis* (meadow salsify) and *Rumex acetosa* (common sorrel) and their effect on proliferation of HaCaT cells. Antiproliferative effects were evaluated *in vitro* using the following concentrations of phenolic compounds in cultivation medium: 100, 75, 50 and 25 µg/mL. Phenolic composition was also determined by HPLC. The results indicate that even low concentrations of these flowers’ phenolic compounds inhibited cell proliferation significantly and the possible use of the studied herb’s flowers as sources of active phenolic compounds for human nutrition.

## 1. Introduction

Phenolic compounds (PhC) and their anti-tumour effects have been studied for many years [1]. Grape seeds and skins [2], tea [3] or fruits [4,5] are considered to be rich on these phytochemicals. Every plant not only has different concentrations of PhC, but their composition and content in every part is different [6]. Researchers’ attention in terms of effects on tumour diseases has been mostly focused on wine PhC [7] or tea PhC [8], but the effect of herb flowers, which are also good source of phytochemicals [9], has not been described yet. In the present study the plants *Allium schoenoprasum* (chive) *Rumex acetosa* (common sorrel) and *Tragopogon pratensis* (meadow salsify) which could be easily available sources of PhC in Europe were studied for the first time in the context of their potential anti-tumour effects.

PhC constitute a heterogeneous class of compounds [10] with varied protective effects [3,11]. PhC have been reported to display a variety of biological actions. They can act as antioxidants [12], antiangiogenics [13], selective estrogen receptor modifiers [14], anti-carcinogenic and anti-inflammatory agents [15] and many others. The most significant properties of PhC that may affect carcinogenesis are trapping of ultimate carcinogens [16], inhibitory action against nitrosation reactions [6], inhibition of cell proliferation-related activities [17], induction of cell apoptosis [16], cell cycle arrest [18], blockade of mitotic signal transduction through modulation of growth factor receptor binding [16], nuclear oncogene expression [19], inhibition of DNA synthesis [20] and modulation of signal transduction pathways by altered expression of key enzymes such as cyclooxygenases and protein kinases [21]. The aim of this study is to determine the effect of PhC contained in the flowers of three herb species on cell proliferation and to demonstrate the suitability of this herbs for the prevention of tumour diseases.

## 2. Results and Discussion

Several hundred different PhC have been identified in plants [22]. In this study the following ten PhC were detected by HPLC: gallic acid (**GA**), coumaric acid (**CA**), ferulic acid (**FA**), rutin (**Ru**), resveratrol (**Re**), vanillic acid (**VA**), sinapic acid (**SA**), catechin (**C**), quercetin, caffeic acid (**CA**) and cinnamic acid. The herb flowers used in this study (*A. schoenoprasum*, *T. pratensis and R. acetosa*) did not contain all of these PhC. Although quercetin is one of the most common flavonoids in plants, it was not detected in any of the studied herbs. No cinnamic acid was found either.

The content of PhC in dry matter of the studied herb flowers is shown in Table 1. *A. schoenoprasum* contains **GA** (201.76 µg/g), **CA** (207.29 µg/g), **FA** (887.44 µg/g) and **Ru** (20.26 µg/g). Most types of PhC were determined in *T. pratensis*. These were **GA** (1347.85 µg/g), **FA** (197.79 µg/g), **Ru** (89.99 µg/g), **Re** (13.95 µg/g), **SA** (110.85 µg/g) and **CA** (278.72 µg/g). In *R. acetosa* four kinds of PhC were found: **Re** (41.27 µg/g), **VA** (130.29 µg/g), **SA** (5708.48 µg/g) and **C** (75.46 µg/g).

In this study HaCaT cells were used to determine antiproliferative activity. As can be seen from Table 2, cells incubated in the presence of extracts have remarkable lower proliferation compared with control. These differences are statistically significant (Table 2).

**Table 1:** Content of phenolic compounds in herbs.

PC		*Allium schoenoprasum*		*Tragopogon pratensis*		*Rumex acetosa*	
		Extract	Dry matter	Extract	Dry matter	Extract	Dry matter
		(µg/mL)	(µg/g)	(µg/mL)	(µg/g)	(µg/mL)	(µg/g)
**GA**	Gallic acid	8.45	201.76	66.16	1347.85	/	/
**CA**	Coumaric acid	8.50	207.29	/	/	/	/
**FA**	Ferulic acid	37.16	887.44	9.71	197.79	/	/
**Ru**	Rutin	0.85	20.26	4.39	89.99	/	/
**Re**	Resveratrol	/	/	0.68	13.95	3.23	41.27
**VA**	Vanillic acid	/	/	/	/	11.03	130.29
**SA**	Sinapic acid	/	/	5.28	110.85	483.21	5708.48
**C**	Catechin	/	/	/	/	6.39	75.46
**CA**	Caffeic acid	/	/	13.68	278.72	/	/

**Table 2:** Antiproliferation effect of different concentration of herb flowers’ phenolic compounds on HaCaT cells quantified by a MTT assay (Average absorbance ± SD).

*Allium schoenoprasum* 25 µg/mL	0.1975 ± 0.0128 **
*Allium schoenoprasum* 50 µg/mL	0.2043 ± 0.0253 **
*Allium schoenoprasum* 75 µg/mL	0.2151 ± 0.0164 **
*Allium schoenoprasum* 100 µg/mL	0.1930 ± 0.0221 **
*Rumex acetosa* 25 µg/mL	0.5873 ± 0.0671 **
*Rumex acetosa* 50 µg/mL	0.4472 ± 0.0643 **
*Rumex acetosa* 75 µg/mL	0.2367 ± 0.0578 **
*Rumex acetosa* 100 µg/mL	0.1903 ± 0.0203 **
*Tragopogon pratensis* 25 µg/mL	0.4090 ± 0.0216 **
*Tragopogon pratensis* 50 µg/mL	0.1991 ± 0.0433 **
*Tragopogon pratensis* 75 µg/mL	0.1666 ± 0.0104 **
*Tragopogon pratensis* 100 µg/mL	0.1738 ± 0.0165 **
Control	0.8187 ± 0.1806 *

Note: Values with different superscripts show significance level within column: P < 0.01 (*,**).

**Figure 1:**
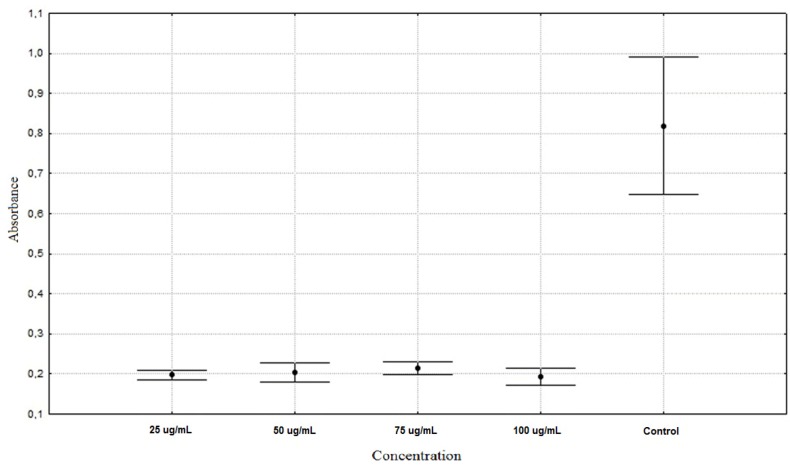
Antiproliferation activity of *Allium schoenoprasum* phenolic compounds on HaCaT cells (average ± SD).

Figure 1 shows the antiproliferation activity of *A. schoenoprasum* extracts The most abundant PhC in *A. schoenoprasum* was **FA** (Table 1), which is one of the most common phenolic acids in plants. For example, content of **FA** in lavender is 5.3 µg/g dry sample [23], in Crete oregano 3.4 µg/g dry sample and in mountain tea 69.5 µg/g dry sample [24]. **FA** has many biological activities like improvement of microcirculation, elimination of oxygen-free radicals, anti-inflammatory properties [25] and suppression of carcinogenesis [26]. According to Lin *et al*. [25], FA has the ability to inhibit cellular proliferation and tumour development, which matches our results. **GA**, **CA** and **Ru** were also detected in *A. schoenoprasum*, but their content was rather low.

**FA** is also found in *T. pratensis*, but the content is nearly four times lower than in *A. schoenoprasum*. *T. pratensis* also contained **GA**, **Ru**, **Re**, **SA** and **CA**. The PhC of highest concentration in *T. pratensis* was found to be **GA** (1,347.85 µg/g). According to Proestos *et al*. [24] the content of **GA** is, for example, 15 µg/g dry sample in eucalyptus and 26 µg/g dry sample in mountain tea. **GA** is a free radical scavenger with significant inhibitory effects on cell proliferation, it induces apoptosis in a series of cancer cell lines, and shows selective cytotoxicity against tumour cells with higher sensitivity than normal cells [27,28].

**Figure 2:**
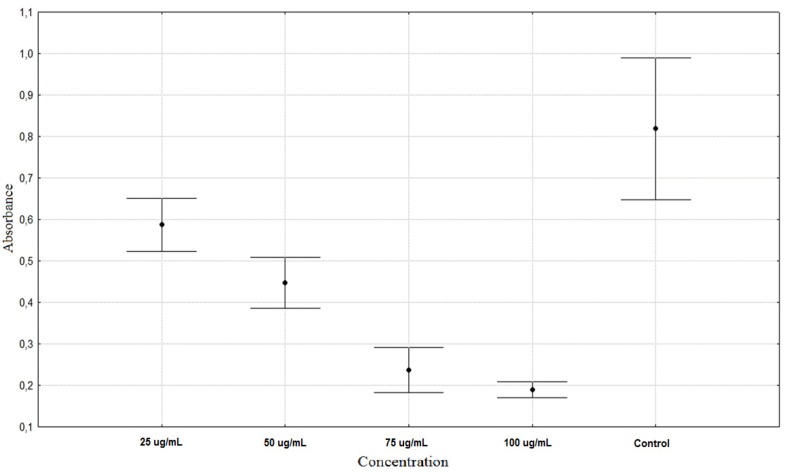
Antiproliferation activity of *Rumex acetosa* phenolic compounds on HaCaT cells (average ± SD).

**Figure 3:**
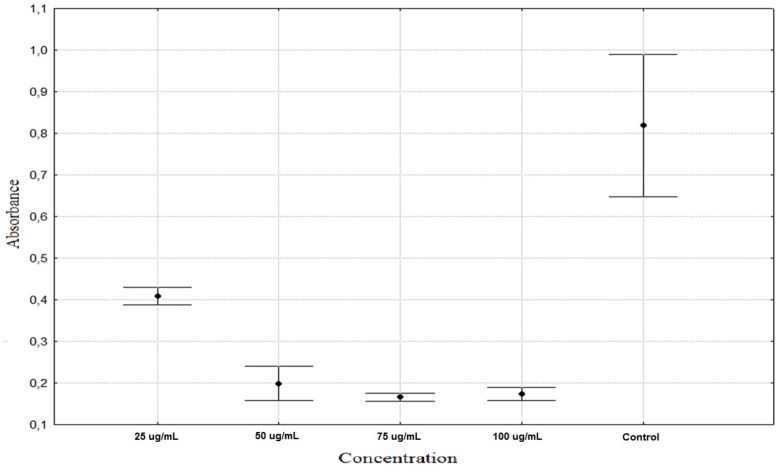
Antiproliferation activity of *Tragopogon pratensis* phenolic compounds on HaCaT cells (average ± SD).

In contrast to *A. schoenoprasum,* extracts of *T. pratensis* and *R. acetosa* decreased the proliferation gradually. However the differences between each concentration and control were statistically significant in all cases (Table 2). *R. acetosa* shows similar antiproliferation activity at concentrations of 75 and 100 µg/mL (Figure 2). *T. pratensis* shows similar activity at PhC concentrations of 50, 75 and 100 µg/mL (Figure 3).

*R. acetosa* contained **Re**, **VA**, **SA** and **C**. The most abundant PhC was **SA** (5,708.48 µg/g). Extracts from *R. acetosa* had the lowest antiproliferation activity (Table 2), which can be caused by a phenomena described and explained by Kampa *et al*. [29] whereby the shortening of the side chain in SA leads to a loss of the antiproliferative activity.

PhC extracted from the herbs used for this study have higher antiproliferative activity in comparison with PhC used in other studies. For example, black tea PhC at a concentration of 100 µg/mL reduced cell viability by 60% [30]. Different camellia flower extracts at the same concentration decrease the cell viability in the range from 10 to 60% [31]. Results in this study reached values of about 80% decreased cell viability. These different results could be caused by different times of incubation and the use of different cell lines, which may be more toxicity resistant, as Murugan *et al*. [30] used HepG2 cells and Way *et al*. [31] used MCF-7 cells.

The observed antiproliferative activity of PhC can be explained by their modulation of different key targets of pathways controlling cell proliferation, differentiation, expression and cell death. The MAPK pathways can be used as example [32,33]. They include extracellular signal-regulated kinase (ERK), c-Jun Nterminal Kinase (JNK) and p38 MAPK [34]. According to Yeh and Yen [34] **GA**, which is present in *T. pratensis* and in *A. schoenoprasum*, increased the levels of phosphorylated JNK and p38 and almost completely blocked inhibition of the p38 MAPK pathway. *T. pratensis* and *A. schoenoprasum* also contain **FA**, which inhibits the activation of ERK [35]. JNK and p38 MAPK are also activated by **Re**, indentified in *R. acetosa* and *T. pratensis* [33]. SA, present in very high amounts in *R. acetosa* and also found in *T. pratensis*, is involved in the MAPK pathways too [36]. Another signal molecule affected by PhC is Activator protein 1 (AP-1). For example, Re blocks AP-1-mediated gene expression [37]. **GA** and **C** inhibit AP-1 binding activity [38]. Other PhC like **FA**, **SA** and **CA** also have effects on AP-1 [36,39]. These PhC were present in every one of the three studied herb flowers.

**Figure 4:**
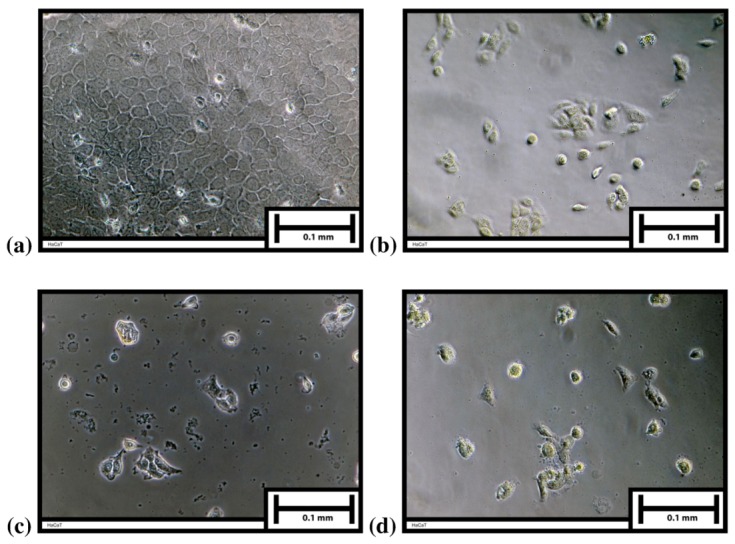
**(a)** Control; **(b)** HaCaT cells incubated in presence of *Allium schoenoprasum* extract (75 µg/mL); **(c)** HaCaT cells incubated in presence of *Rumex acetosa* extract (75 µg/mL); **(d)** HaCaT cells incubated in presence of *Tragopogon pratensis* extract (75 µg/mL).

Figure 4 shows differences between morphology of control [Figure 4(a)] and HaCaT cells incubated in the presence of *A. schoenoprasum* PhC [Figure 4(b)], *R. acetosa* extract [Figure 4(c)] and *Tragopogon pratensis* extract [Figure 4(d)] at a concentration of 75 µg/mL, because at this concentration the differences in morphology are best illustrated.

This study has demonstrated the impact of herbal flowers PhC on the proliferation of HaCaT cells. The antiproliferative activity depends on each particular herb. In the case of *A. schoenoprasum* the activity was independent of the applied concentration of PhC, as similar activity was observed for all concentrations. The antiproliferative activity of *R. acetosa* and *T. pratensis* varied with the concentration of PhC. In the case of *T. pratensis*, concentrations higher than 50 µg/mL do not have an significant impact on proliferation. In the case of *R. acetosa*, the critical concentration was found to be 75 µg/mL. The different antiproliferative activities of herb extracts can be caused by variable PhC content and composition. Another factor which must be considered is the fact that this study only examined 10 types of polyphenols.

## 3. Experimental

### 3.1. Extraction Conditions

PhC were extracted from flowers of *Allium schoenoprasum*, *Rumex acetosa* and *Tragopogon pratensis*. All flowers were cut during the year 2010 in the Czech Republic in central Europe. Immediately after cutting the flowers were frozen and stored at −40 °C. The extraction was performed according to Hakimuddin *et al*. [40] with some modifications: frozen herb flowers were homogenized in 90% methanol (2 mL/g) and subsequently extracted at 4 °C for 30 minutes. After extraction centrifugation at 1,990 rpm for 10 minutes was used to separate the supernatant. Sediments were subjected to a new extraction. This process was repeated three times. The methanol was removed using a Laborota 4011 digital rotary evaporator (Heidolph, Schwabach, Germany). Subsequently extracts concentration was adjusted to obtain concentration of 1,000 mg/mL. 

### 3.2. Cell Cultivation

The human immortalized non-tumorigenic keratinocyte cell line (HaCaT) [41] supplied by Cell Lines Service (Catalog No. 300493, Eppelheim, Germany) was used. Dulbecco’s Modified Eagle Medium (DMEM) – high glucose, with added fetal bovine serum (10%) and penicillin/streptomycin (100 U/mL) (100 μg/mL) (PAA Laboratories GmbH, Pasching, Austria) was used as the culture medium.

### 3.3. Antiproliferation Test

The PhC extracts were diluted in culture medium (DMEM) to obtain dilutions with concentrations of 100, 75, 50 and 25 μg of PhC per mL of cultivation medium. All dilutions were used immediately. Cells were pre-cultivated for 24 hrs and the culture medium was subsequently replaced by dilutions. As a control experiment, pure medium without PhC was used. To assess antiproliferative activity on HaCaT cells, the MTT assay (Invitrogen Corporation, Carlsbad, California, USA) [42] was performed after three-day cultivation in dilutions. The absorbance was measured at 540 nm using a Sunrise microplate absorbance reader (Tecan, Männedorf, Switzerland). The cell proliferation expressed as MTT absorbance measured in respective dilutions relative to control is presented. All the tests were performed in quadruplicate. The photomicrographs were taken using an inverted Olympus CKX41 phase contrast microscope (Olympus, Hamburg, Germany). The differences between observed absorbance were detected by T-Test using Statistica for Windows.

### 3.4. Determination of PhC

A standard solution of tannin was prepared from tannin (50 mg) dissolved in water (100 mL). The standard solution of tannin was added using a pipette to six 50 mL flasks in volumes of 0.2, 0.3, 0.4, 0.5 mL. Extract (1 mL) was added to the seven flasks and dissolved as needed. Distilled water (20 mL) and the Folin-Ciocalteu reagent (1 mL) was added to every flask. After three minutes 20% solution Na_2_CO_3_ (5 mL) was added. The solutions were mixed and the distilled water was added to a volume of 50 mL. After 30 minutes the color intensity compared to control (no tannin) was measured at 700 nm.

### 3.5. Chromatography

Determination of individual PhC was carried out using a Dionex UltiMate 3000 high performance liquid chromatography (HPLC) system (Dionex, Sunnyvale, California, USA). A Supelcosil LC-18-DB (25 cm × 4.6 mm I.D., S-5 μm) column was used. PhC were detected with DAD UV-Vis detection at 205 nm. The mobile phases used for gradient HPLC elution were: (A) 5% (v/v) acetonitrile, 0.035% (v/v) trifluoroacetic acid and (B) 50% (v/v) acetonitrile, 0.025% (v/v) trifluoroacetic acid. The flow-rate was set at 1.0 mL/min. The gradient elution profile started with A-B (90:10), then B was gradually increased to 20% at 10 min, to 40% at 16 min, to 50% at 20 min and back to 40% from 25 to 27 min [43]. The data presented are the average values calculated from three measurements.

## 4. Conclusions

This study is the first study on the antiproliferation activity of chosen phenolic compounds contained in several herb flowers. The results in this study suggest that the tested herbs are a good source of phenolic compounds and that their concentration and composition varies with each species. The work presented proved that the phenolic compounds contained in medical herbs significantly decrease cell proliferation. The fact that natural phenolic compounds contained in herb flowers (*A. schoenoprasum*, *T. pratensis* and *R. acetosa*) inhibit cell proliferation makes those herb flowers potentially useful for the treatment and prevention of tumour diseases. The results suggest that antiproliferation activity does not depend exclusively on total phenolic compound content or composition, but it can be also influenced by other extracted active substances which were not detected.
